# Physiologic phenotypes in blunt thoracic aortic injury: implications for risk stratification and surgical decision-making using machine learning

**DOI:** 10.3389/fdgth.2026.1745987

**Published:** 2026-02-09

**Authors:** Phillip D. Jenkins, Michael R. Kolesnikov, Shelby Willis, Victor Andujo, Tiffany Lian, Ruchi Thanawala, Castigliano Bhamidipati, Justin Regner, Julie Doberne

**Affiliations:** Department of Surgery, Oregon Health & Science University, Portland, OR, United States

**Keywords:** aortic injury, BTAI, machine learning (ML), physiologic phenotyping, trauma

## Abstract

**Introduction:**

Blunt thoracic aortic injury (BTAI) is rare but highly lethal. Despite advances such as thoracic endovascular aortic repair (TEVAR), management remains challenging due to heterogeneous physiologic presentations. Injury grade alone may not capture early derangements influencing risk. In other acute syndromes, machine-learning–derived physiologic phenotypes have informed prognosis. We applied this approach to BTAI to identify phenotypes, examine outcomes, and use explainable AI (XAI) to characterize their physiologic drivers.

**Methods:**

We analyzed 1,375 patients from the Aortic Trauma Foundation registry using eleven admission physiologic and injury severity variables. After plausibility checks and median imputation, K-means clustering (*k* = 3; silhouette = 0.23) defined physiologic phenotypes. Patients missing >50% physiologic data were excluded; the remaining 1,083 were assigned to the nearest phenotype centroid. Outcomes included in-hospital mortality, treatment modality, hospital and ICU length of stay (LOS), ventilator days, and time to repair. For XAI, an XGBoost classifier was trained to reproduce K-means labels, and SHapley Additive exPlanations (SHAP) quantified feature importance and stability.

**Results:**

Three phenotypes emerged: “Stable” (*n* = 431; preserved hemodynamics, low lactate), “Shock” (*n* = 398; hypotension, tachycardia, high lactate), and “Neurologically Compromised” (*n* = 254; depressed Glasgow Coma Score with intermediate hemodynamics). Mortality was 7.2%, 18.9%, and 18.1% (*p* < 0.001), significantly higher in both high-risk groups vs. Stable. TEVAR predominated (58.2–63.0% across phenotypes); open repair was uncommon (≤6.3%), and medical management was more frequent in Stable (31.1% vs. Shock 19.1%; *p* = 0.0010). ICU LOS differed overall (*p* = 0.008), with Neurologically Compromised requiring longer stays. The XGBoost surrogate reproduced K-means phenotypes with high fidelity (accuracy 0.917; macro-F1 0.906; *κ* 0.873; adjusted Rand index 0.788). SHAP identified systolic blood pressure, lactate, and heart rate as dominant phenotype-defining features, with stable rankings across 200 bootstrap refits.

**Conclusion:**

Machine learning identified three physiologic BTAI phenotypes with distinct presentations and mortality despite similar management patterns. XAI showed that perfusion and metabolic markers, not anatomy alone, drive phenotype structure. These data reinforce the potential for physiologic phenotyping to enhance prognostication and guide BTAI decision-making.

## 1 Introduction

Blunt thoracic aortic injury (BTAI) is relatively rare (approximately 1%) (1) among the most lethal injuries encountered in trauma, second only to traumatic brain injury in mortality among patients with blunt mechanisms (2). While early mortality is common, up to 80% of patients die before reaching the hospital, those who survive to admission still face high risks, particularly when management is delayed or misdirected (2, 3). Advances in imaging and widespread adoption of thoracic endovascular aortic repair (TEVAR) have significantly improved outcomes, reducing in-hospital mortality from historic levels of 40%–50% to below 10% in contemporary cohorts (4). Alongside this progress, large trauma registries such as the Aortic Trauma Foundation (ATF) have improved our understanding of real-world practice patterns (5, 6), highlighting growing reliance on nonoperative management for lower-grade injuries and confirming TEVAR as the standard for anatomically suitable lesions (4–6).

However, prognostication in BTAI remains challenging. Existing approaches rely heavily on anatomic grading systems, such as the Society for Vascular Surgery (SVS) scale derived from the Abbreviated Injury Scale (AIS), which stratifies injuries from intimal tears (grade I) to free rupture (grade IV) (7). While higher grades are generally associated with worse outcomes, anatomic severity alone incompletely captures risk. Several studies have shown that patients with the same injury grade may have markedly different trajectories depending on concomitant injuries, hemodynamic status, and metabolic derangements at presentation (7, 8). Even recent registry-based mortality prediction tools, including those from the Aortic Trauma Foundation, incorporate physiologic parameters alongside injury grade, underscoring that anatomy alone is insufficient (5).

Furthermore, outcomes stratified solely by BTAI grade demonstrate substantial overlap: patients with low-grade injuries may still deteriorate or progress radiographically (9), while select high-grade injuries can be successfully managed nonoperatively (10, 11). This variability reflects the influence of early physiologic instability, such as hypotension, elevated lactate, or depressed Glasgow Coma Score, which are not captured in anatomic classification systems. Current prognostic frameworks thus provide only a partial view, and no widely adopted tool integrates standardized physiologic profiling into risk assessment for BTAI.

In critical care and trauma literature, there has been growing recognition that early physiologic variables, such as hypotension, tachycardia, lactate burden, and base deficit, can be used to define clinically meaningful subgroups, or physiologic phenotypes, that correlate with prognosis and guide treatment (12, 13). In syndromes like sepsis, acute respiratory distress syndrome (ARDS), and traumatic brain injury (TBI), unsupervised machine learning has been used to identify phenotypes associated with differential outcomes and even treatment response (12–14). These approaches have led to a more nuanced, personalized understanding of these heterogeneous conditions (15, 16).

Despite the clear physiologic variability seen in BTAI, similar phenotyping approaches have not been applied in this injury pattern. Prior BTAI studies have largely focused on anatomical injury grading or procedural outcomes, without leveraging early physiologic data for stratification (5, 17). The potential to define risk-relevant subgroups using presenting vital signs and labs remains unexplored in the BTAI population.

To our knowledge, this is the first study to apply unsupervised machine learning to identify physiological phenotypes in BTAI. Using real-world data from the Aortic Trauma Foundation registry, we aimed to uncover reproducible subgroups based on standardized physiologic features available at presentation. Our goal was twofold: (1) to identify data-driven phenotypes using clustering techniques, and (2) to evaluate the relationship between these phenotypes and key clinical outcomes including in hospital mortality, hospital and ICU length of stay, ventilator days, and treatment strategy (medical management with anti-impulse therapy, TEVAR, or open surgical repair).

By identifying actionable physiological phenotypes at the time of hospital arrival, we seek to provide a framework for risk stratification, surgical decision-making, and resource triage that goes beyond injury grade. This phenotypic approach represents a novel and potentially impactful method for improving the care of patients with BTAI.

## 2 Methods

### 2.1 Study population and data source

We used the Aortic Trauma Foundation (ATF) Blunt Thoracic Aortic Injury (BTAI) registry, which includes detailed demographic, injury, physiologic, imaging, management, and outcome variables for patients with confirmed BTAI. The source dataset contained 1,375 patients. For the primary analysis cohort, we excluded patients missing more than 50% of the prespecified physiologic variables (defined below) to limit over-imputation from sparse records; 1,083 patients remained for analysis.

### 2.2 Physiologic variables and preprocessing

Phenotyping targeted eleven admission variables selected *a priori* to capture early hemodynamic, neurologic, metabolic, and injury-burden signals: systolic blood pressure (SBP), heart rate (HR), Glasgow Coma Score (GCS), temperature, lactate, base deficit value, pH, creatinine, hemoglobin, platelet count, and Injury Severity Score (ISS). Data were inspected for plausibility and harmonized. To preserve true extremes seen during resuscitation, we allowed SBP and HR values down to 0 (e.g., transient loss of pulses) and used wide physiologic bounds for other labs (e.g., lactate up to 50 mmol/L). Non-physiologic entries and unit inconsistencies (e.g., platelet counts recorded in thousands) were corrected when resolvable or set to missing if not. After excluding patients with >50% missing across physiologic variables, remaining missing values were imputed by variable-wise medians and all features were standardized (z-scores) for modeling.

### 2.3 Derivation of physiologic phenotypes

We first performed unsupervised clustering on the full 1,375-patient cohort using K-means clustering, an unsupervised machine learning algorithm that partitions observations into a prespecified number of clusters by minimizing within-cluster variance. This approach groups patients with similar multivariable physiologic profiles without using outcome information and has been widely applied in clinical phenotyping studies across trauma and critical care populations (12–14).

To determine the appropriate number of clusters, we evaluated solutions ranging from *k* = 2 to *k* = 6 and selected *k* = 3 based on silhouette score optimization (silhouette = 0.23). The silhouette score quantifies how similar each observation is to its assigned cluster relative to other clusters, with values ranging from −1 to 1; higher values indicate better separation, whereas values near zero suggest overlap. In complex physiologic datasets with overlapping clinical features, modest silhouette values are common and do not preclude clinically meaningful subgroup identification.

The three clusters were labeled *post hoc* according to their dominant physiologic characteristics. One cluster demonstrated preserved vital signs and low lactate burden and was labeled “Stable”. A second cluster was characterized by hypotension, tachycardia, and elevated lactate and was labeled “Shock”. The third cluster showed markedly depressed Glasgow Coma Score (GCS) with intermediate hemodynamics and was labeled “Neurologically Compromised.” Principal component analysis (PCA) of standardized physiologic variables was used for visualization only to illustrate separation between clusters and was not used for model derivation.

### 2.4 Centroid-based phenotype assignment for the analysis cohort

Because physiologic clustering is sensitive to missing data patterns and sample composition, we derived phenotype centroids using the full 1,375-patient cohort after imputation, then applied these fixed centroids to a stricter analysis cohort. For the primary analysis, we excluded patients with more than 50% missing physiologic variables, yielding 1,083 patients.

Each patient in this analysis cohort was assigned to the nearest phenotype centroid from the *k* = 3 derivation model using Euclidean distance in standardized feature space. This centroid-based nearest-neighbor classification approach preserves the original cluster definitions while allowing consistent phenotype assignment in a dataset with reduced missingness. Importantly, this avoids re-running clustering on a smaller or altered cohort, which could result in label drift and compromise interpretability and reproducibility.

This two-step approach, first an unsupervised derivation followed by centroid-based assignment, has been successfully used in other biomedical domains, including vaginal microbiota classification and population health phenotyping, to enable reproducible application of data-driven phenotypes across cohorts (18–20).

### 2.5 Outcomes and definitions

The primary outcome was in-hospital mortality. Secondary outcomes included treatment modality [medical management, thoracic endovascular aortic repair (TEVAR), open surgical repair], hospital length of stay (LOS), intensive care unit (ICU) LOS, ventilator days, and time from admission to definitive repair (open or endovascular). When multiple treatment fields were present, mutually exclusive “initial modality” was assigned with a prespecified priority of open repair > TEVAR > medical management; otherwise the record was classified as none/unknown.

### 2.6 Statistical analysis

Categorical variables were compared across physiologic phenotypes using *χ*² tests or Fisher's exact tests when expected cell counts were <5. Continuous variables were summarized as medians with interquartile ranges and compared across phenotypes using one-way analysis of variance (ANOVA) or nonparametric alternatives, as appropriate.

Prior to ANOVA, distributional assumptions were evaluated through visual inspection of residual plots and assessment of variance homogeneity across groups. When deviations from normality or heteroscedasticity were observed, the Kruskal–Wallis test, a rank-based nonparametric alternative to ANOVA, was used to compare distributions across phenotypes. When overall group differences were significant, *post hoc* pairwise comparisons were performed using Dunn's test with Bonferroni correction to control for multiple testing.

In-hospital mortality and treatment modality were analyzed using contingency tables. Overall differences across phenotypes were assessed with *χ*² tests, and when significant, pairwise comparisons were conducted using Fisher's exact tests with Bonferroni adjustment. Fisher's exact test was selected for pairwise analyses because it provides an exact *p*-value and remains valid when cell counts are small, which occurred in several subgroup comparisons (e.g., open repair and none/unknown treatment categories). This approach ensured robust inference while controlling for multiple comparisons.

Time-to-intervention variables exhibited skewed distributions and were analyzed using the Kruskal–Wallis test, followed by Dunn's *post hoc* testing when appropriate. Two-sided *p* values < 0.05 were considered statistically significant after adjustment.

All analyses were performed in Python 3.12.4 (pandas, scikit-learn, scipy, scikit-posthocs), and figures were generated with matplotlib (*Python 3 Reference Manual*. Scotts Valley, CA: CreateSpace). PCA plots depict cluster separation but were not used for modeling.

### 2.7 Explainable artificial intelligence (XAI) analysis

To enhance the interpretability of the unsupervised physiologic clustering, we conducted a *post hoc* explainable artificial intelligence (XAI) analysis using a supervised surrogate model and SHapley Additive exPlanations (SHAP). XAI methods aim to improve transparency of complex machine learning models by quantifying how individual input features contribute to model outputs. The goal was to assess how well a physiologic classifier could reproduce the K-means–derived phenotypes and to quantify the relative contribution of each physiologic variable to phenotype assignment. Importantly, this step was designed to explain the clustering structure rather than to introduce a novel predictive model.

We trained an XGBoost gradient boosting classifier, a tree-based ensemble learning method well-suited for tabular clinical data, on the standardized admission physiologic variables used for clustering: systolic blood pressure, heart rate, Glasgow Coma Score (GCS), temperature, lactate, base deficit, pH, creatinine, hemoglobin, platelet count, and Injury Severity Score (ISS). The outcome was the three-class phenotype label (Stable, Shock, Neurologically Compromised) originally assigned by K-means. The dataset was randomly split 80:20 into training and held-out test sets with stratification by phenotype. Model performance was evaluated on the held-out test set using accuracy, macro–F1 score, Cohen's *κ*, and the adjusted Rand index (ARI), which together quantify classification performance and agreement with the underlying cluster structure beyond chance, to quantify agreement with the underlying cluster structure. Five-fold cross-validation was performed on the training set to reduce optimism from a single data split and to estimate generalizable model fidelity.

Global and local feature attributions were obtained using the SHAP TreeExplainer. SHAP is a game theory–based approach that assigns each feature a contribution value based on its marginal impact on model predictions (21). Global importance was defined as the mean absolute SHAP value for each feature across all patients. Class-specific SHAP beeswarm plots were generated to visualize both the magnitude and direction of each feature's contribution within each phenotype. To assess stability of the feature importance rankings, we performed 200 bootstrap refits of the XGBoost model, recomputed global mean absolute SHAP values for each refit, and calculated pairwise Spearman rank correlations between bootstrap-derived rankings and the original model.

## 3 Results

### 3.1 Cohort characteristics

**Figure 1:**
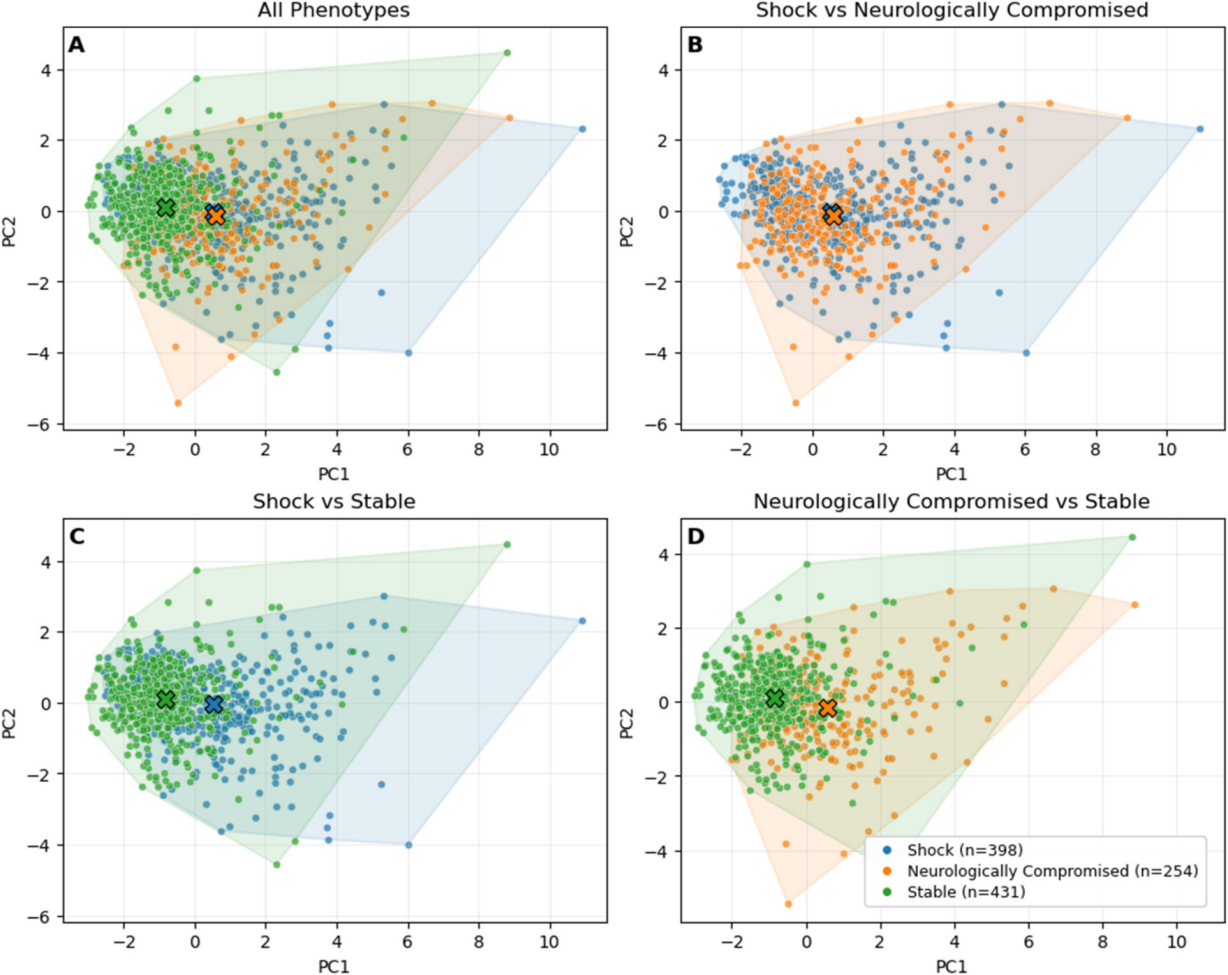
PCA projection of physiologic phenotypes with patients colored by phenotype. **(A)** All phenotypes shown together. **(B)** Shock vs. Neurologically Compromised. **(C)** Shock vs. Stable. **(D)** Neurologically Compromised vs. Stable. X markers indicate the centroid of each phenotype cluster in principal component space.

After exclusions for >50% missing physiologic data, 1,083 patients remained for analysis. The three physiological phenotypes had been defined in the full 1,375-patient derivation cohort using unsupervised clustering. The silhouette score for the three-cluster solution was modest (0.23), reflecting partial overlap between physiologic profiles rather than discrete separation. To classify the 1,083-patient analysis cohort, each patient's physiologic profile was compared with the average profile (“centroid”) of each phenotype from the derivation step, and they were assigned to the closest match. This preserved the original phenotype definitions and allowed consistent classification without re-running the clustering. The resulting groups showed distinct physiologic patterns and in-hospital mortality rates. One group demonstrated preserved vital signs and low lactate (“Stable,” *n* = 431, 39.8%), another had hypotension, tachycardia, and elevated lactate (“Shock,” *n* = 398, 36.7%), and the third had markedly depressed GCS with intermediate hemodynamics (“Neurologically Compromised,” *n* = 254, 23.5%). These descriptive labels were assigned *post hoc* by the investigators to reflect the defining characteristics of each group. Principal component analysis (PCA) of standardized physiologic variables demonstrated clear visual separation of the three phenotypes (Figure 1).

Baseline demographic, injury, and physiologic characteristics stratified by phenotype are summarized in Table 1. The three groups differed significantly across multiple admission physiologic variables, including systolic blood pressure, heart rate, lactate, Glasgow Coma Score, and Injury Severity Score (all *p* < 0.001), consistent with their descriptive phenotype labels. The “Stable” phenotype demonstrated preserved hemodynamics and lower injury burden, whereas the “Shock” phenotype exhibited hypotension, tachycardia, and the highest lactate levels. The “Neurologically Compromised” phenotype showed markedly depressed neurologic status with intermediate hemodynamic derangements and the highest overall injury severity.

**Table 1:** Demographics and baseline characteristics by physiologic phenotype.

Variable	*N*	Stable	Shock	Neurologically Compromised	*P* value
Age	1,083	41.0 (29.0–58.5)	39.0 (28.0–54.0)	39.0 (29.0–53.0)	0.1438
Injury Severity Score	934	29.0 (24.0–38.0)	34.0 (24.0–43.0)	36.0 (29.0–43.0)	**0** **.** **0000**
Systolic blood pressure	1,075	131.0 (118.0–145.5)	101.0 (80.0–128.0)	102.5 (90.0–115.0)	**0** **.** **0000**
Heart Rate	1,073	88.0 (78.0–100.0)	110.0 (90.0–129.0)	104.5 (93.0–117.0)	**0** **.** **0000**
Lactate	851	2.8 (1.9–4.0)	5.3 (3.7–7.6)	3.8 (2.8–5.0)	**0** **.** **0000**
Glasgow Coma Score	1,068	15.0 (12.5–15.0)	14.0 (3.0–15.0)	14.0 (3.0–15.0)	**0** **.** **0000**
Gender	1,083				0.1482
Female		95 (22.0%)	91 (22.9%)	72 (28.3%)	
Male		336 (78.0%)	307 (77.1%)	182 (71.7%)	
Smoker	1,083				**0** **.** **0172**
No		350 (81.2%)	337 (84.7%)	227 (89.4%)	
Yes		81 (18.8%)	61 (15.3%)	27 (10.6%)	
Hypertension	1,083				**0** **.** **0017**
Absent		321 (74.5%)	334 (83.9%)	209 (82.3%)	
Present		110 (25.5%)	64 (16.1%)	45 (17.7%)	
Coronary Artery Disease	1,083				**0** **.** **0335**
Absent		408 (94.7%)	390 (98.0%)	246 (96.9%)	
Present		23 (5.3%)	8 (2.0%)	8 (3.1%)	
Mechanism	1,081				0.1641
Auto vs. Pedestrian		37 (8.6%)	56 (14.1%)	35 (13.8%)	
Fall		34 (7.9%)	30 (7.5%)	22 (8.7%)	
Motor vehicle collision		266 (61.7%)	234 (58.8%)	149 (58.7%)	
Motorcycle accident		65 (15.1%)	60 (15.1%)	40 (15.7%)	
Other		25 (5.8%)	16 (4.0%)	7 (2.8%)	
Work-related accident		4 (0.9%)	1 (0.3%)	0 (0.0%)	
Hospital transfers	1,082				**0** **.** **0377**
No		416 (96.5%)	393 (98.7%)	250 (98.4%)	
Yes		15 (3.5%)	4 (1.0%)	4 (1.6%)	

Values are median (interquartile range) for continuous variables and *n* (%) for categorical variables. Continuous variables were compared using the Kruskal–Wallis test due to non-normal distributions, and categorical variables were compared using *χ*² tests.

Bold values denote variables with statistically significant differences across physiologic phenotypes (*p* < 0.05).

Demographic characteristics were largely similar across phenotypes, though modest differences were observed in smoking status, hypertension prevalence, coronary artery disease, and interhospital transfer rates (Table 1). These findings indicate that the observed phenotypic separation was driven primarily by physiologic and injury-related variables rather than baseline demographic differences.

### 3.2 Stable phenotype (*n* = 431, 39.8%)

Patients in the “Stable” group had preserved hemodynamics [median SBP 131.0 [118.0–145.5] mmHg, HR 88.0 [78.0–100.0] bpm] and low lactate [2.8 (1.9–4.0) mmol/L]. Neurologic status was generally normal [median GCS 15.0 (12.5–15.0)]. This group had the lowest injury burden [median ISS 29.0 (24.0–38.0)] and the highest prevalence of hypertension (25.5%). Smoking was reported in 18.8%, and coronary artery disease in 5.3%.

### 3.3 Shock phenotype (*n* = 398, 36.7%)

**“**Shock” patients were defined by hypotension [SBP 101.0 (80.0–128.0) mmHg], tachycardia [HR 110.0 (90.0–129.0) bpm], and the highest lactate levels [5.3 (3.7–7.6) mmol/L]. Injury severity was greater than in Stable patients [ISS 34.0 (24.0–43.0)]. Hypertension was less common (16.1%), and smoking prevalence was 15.3%. Coronary artery disease was rare (2.0%).

### 3.4 Neurologically compromised phenotype (*n* = 254, 23.5%)

This group was characterized by markedly depressed neurologic function [median GCS 14.0 (3.0–15.0)] with intermediate hemodynamics [SBP 102.5 [90.0–115.0] mmHg, HR 104.5 [93.0–117.0] bpm] and moderate lactate elevation [3.8 (2.8–5.0) mmol/L]. Injury severity was highest overall [ISS 36.0 (29.0–43.0)]. Hypertension prevalence was 17.7%, smoking was least common (10.6%), and coronary artery disease occurred in 3.1% of patients.

### 3.5 In-hospital mortality

**Figure 2:**
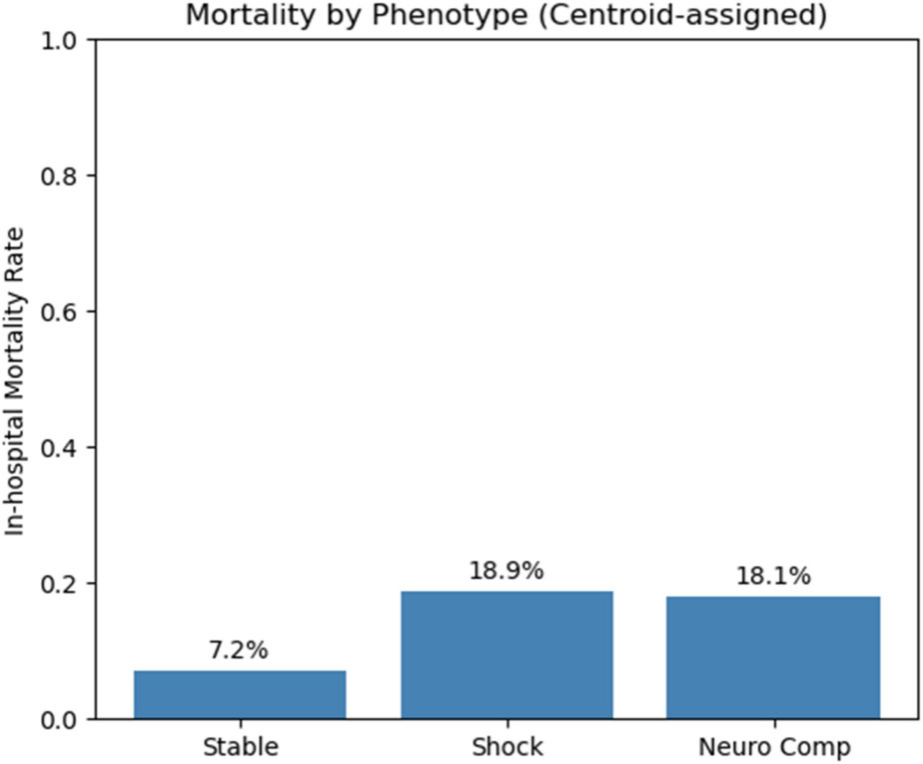
Bars represent the proportion of patients who died during the index hospitalization within each physiologic phenotype (stable, shock, neurologically compromised).

Mortality differed significantly by phenotype (*χ*² = 27.821, *p* < 0.001). Observed mortality was 7.2% in “Stable” (30/431), 18.9% in “Shock” (72/398), and 18.1% in “Neurologically Compromised” (46/254) (Figure 2). Pairwise Fisher's exact tests with Bonferroni correction demonstrated significantly higher mortality in both “Shock” vs. “Stable” (adj. *p* = 0.000004) and “Neurologically Compromised” vs. “Stable” (adj. *p* = 0.000041), with no difference between “Shock” and “Neurologically Compromised” (adj. *p* = 1.000).

### 3.6 Treatment modality

**Figure 3:**
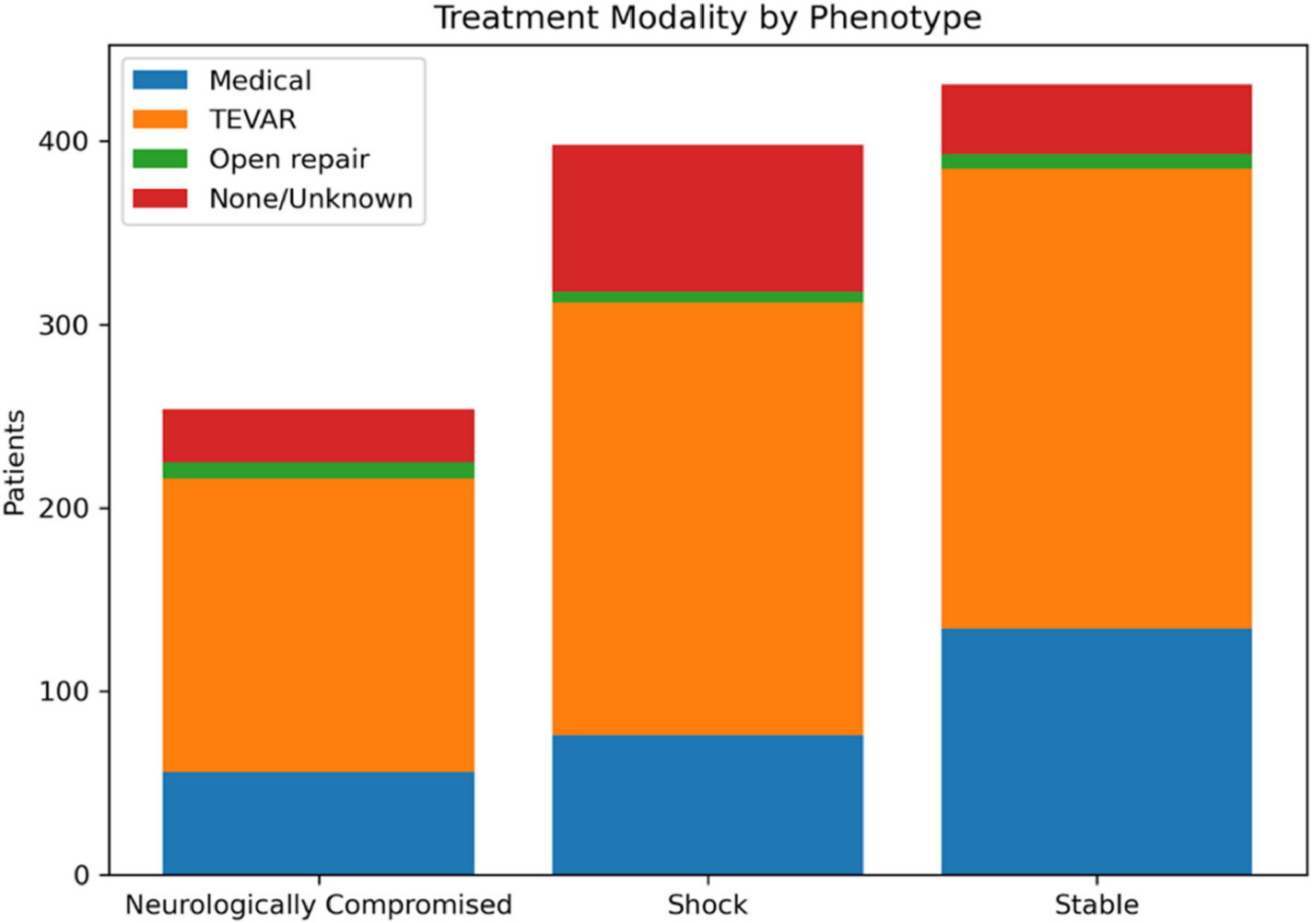
Absolute counts of initial treatment modality [medical management, thoracic endovascular aortic repair (TEVAR), open repair, or none/unknown] stratified by physiologic phenotype. Treatment categories were assigned using a prespecified hierarchy (open repair > TEVAR > medical management).

**Figure 4:**
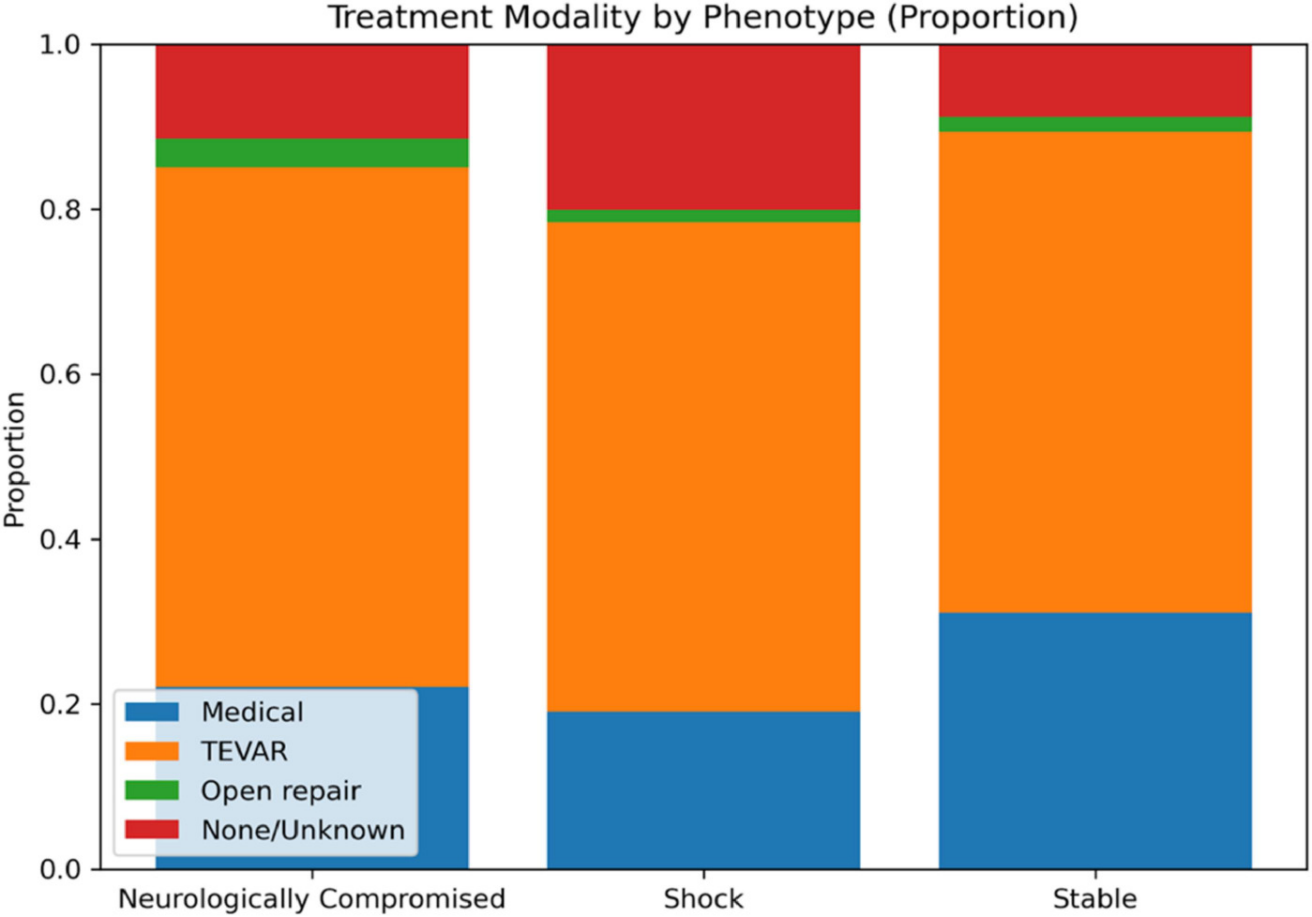
Stacked bar plot showing the relative distribution of treatment modalities within each physiologic phenotype. TEVAR predominated across all phenotypes, while the relative use of medical management and none/unknown treatment varied by physiologic group. Percentages are calculated within phenotype.

Treatment modality varied significantly by phenotype (*χ*² = 37.415, *p* < 0.001), with TEVAR being the most frequent intervention across all groups but with differing relative distributions. “Stable” patients most often underwent TEVAR (58.2%) or medical management (31.1%), whereas “Shock” and “Neurologically Compromised” patients had lower use of medical management (19.1% and 22.0%, respectively) and higher proportions of “none/unknown” treatment (20.1% and 11.4%, respectively). Pairwise testing confirmed that “Stable” patients were significantly more likely than “Shock” patients to receive medical management (adj. *p* = 0.0010) and less likely to have no documented intervention (adj. *p* < 0.0001). “Shock” patients also had a higher rate of no documented intervention compared with “Neurologically Compromised” patients (adj. *p* = 0.0437) (Figures 3, 4).

Within-phenotype mortality analyses demonstrated strong associations between treatment modality and survival. In the “Shock” phenotype, mortality was highest with open repair (40.0%) and “none/unknown” treatment (33.8%), intermediate with TEVAR (13.7%), and lowest with medical management (15.1%) (*χ*² = 24.745, *p* < 0.0001). In “Neurologically Compromised” patients, mortality was highest with open repair (77.8%) and “none/unknown” treatment (57.1%), compared with much lower mortality after TEVAR (9.8%) or medical management (14.5%) (*χ*² = 56.293, *p* < 0.0001). “Stable” patients had low overall mortality, but deaths were more frequent in the “none/unknown” group (21.6%) compared with TEVAR (7.1%) or medical management (3.7%) (*χ*² = 14.432, *p* = 0.0024).

### 3.7 Hospital and ICU length of stay

**Figure 5:**
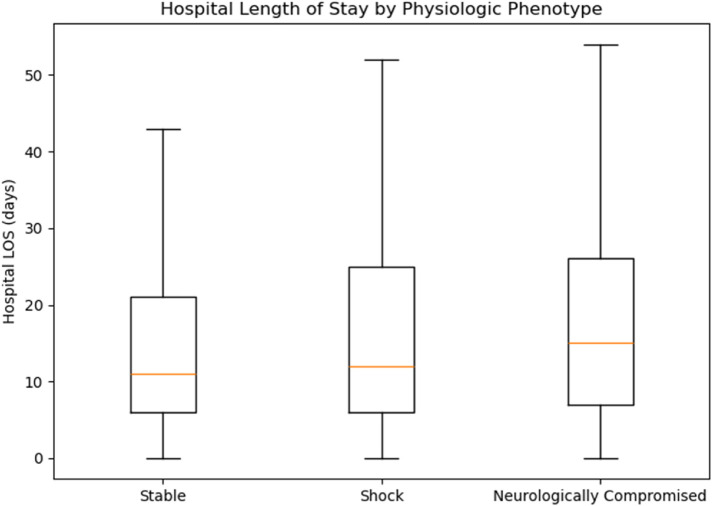
Hospital length of stay by phenotype.

**Figure 6:**
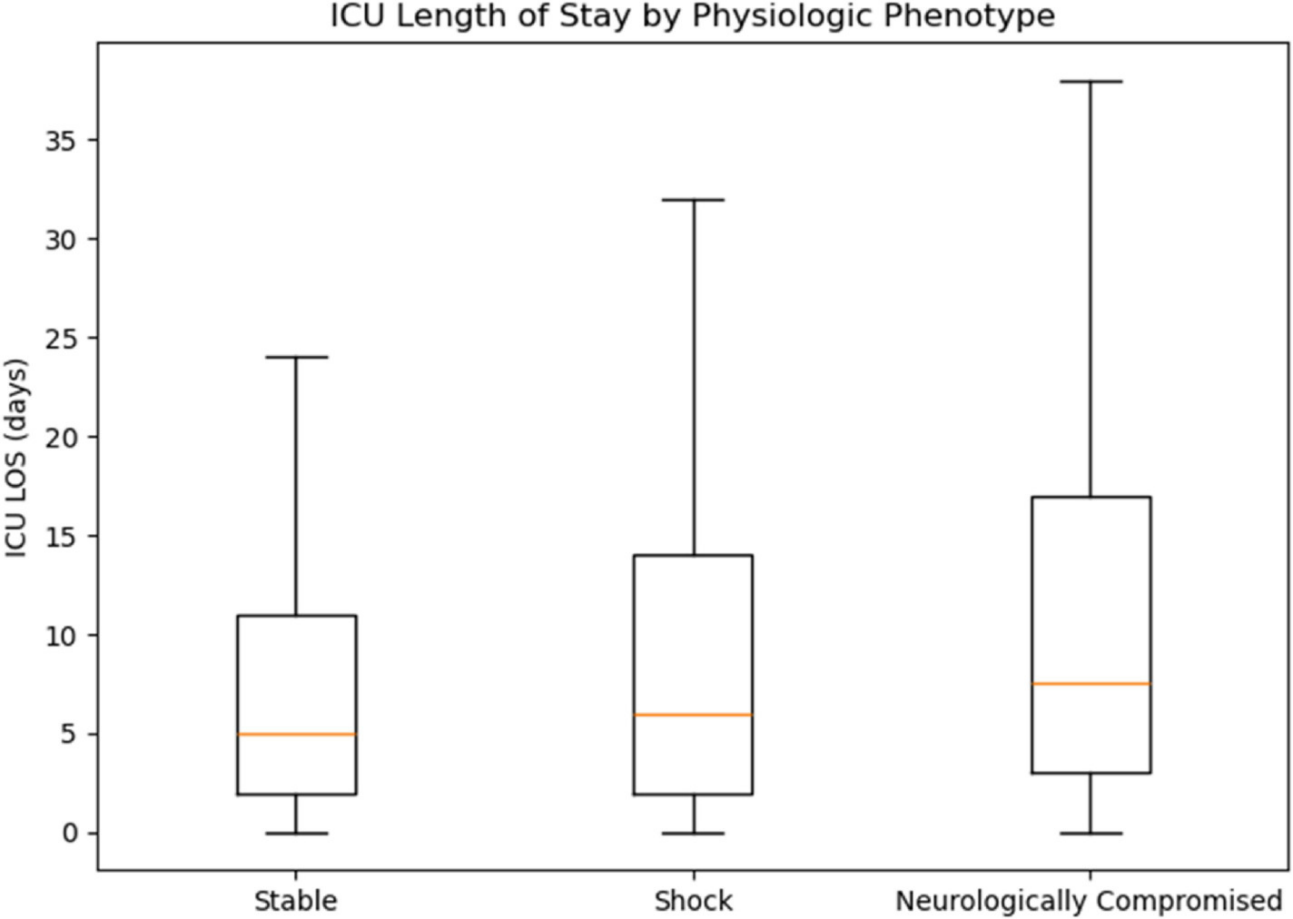
ICU length of stay by phenotype.

Hospital LOS showed a nonsignificant trend across phenotypes (ANOVA F = 2.235, *p* = 0.107). Exploratory *post hoc* testing indicated slightly longer hospital stays in “Neurologically Compromised” vs. “Stable” (Bonferroni-adjusted *p* = 0.0426), whereas differences between “Shock” and the other groups were not significant (Figure 5). ICU LOS differed significantly overall (ANOVA *F* = 4.854, *p* = 0.008); “Neurologically Compromised” required longer ICU care than “Stable” (*p* = 0.000587), with no significant differences between “Shock” and the other groups after adjustment (Figure 6).

### 3.8 Ventilator days

Ventilator days did not differ significantly overall (ANOVA *F* = 1.571, *p* = 0.208). Exploratory *post hoc* comparisons suggested more ventilator days in “Neurologically Compromised” vs. “Stable” (*p* < 0.0001) and in “Shock” vs. “Stable” (*p* = 0.000517); these pairwise findings should be interpreted cautiously given the nonsignificant overall test.

### 3.9 Time to intervention

Time from admission to open repair did not differ significantly across phenotypes (Kruskal–Wallis H = 2.400, *p* = 0.301). Median time to open repair was shortest in the “Neurologically Compromised” group [1.3 h (IQR 1.0–4.5)] compared with “Shock” [8.9 h (0.6–134.7)] and “Stable” [13.0 h (4.5–25.7)], though pairwise Dunn's tests with Bonferroni correction found no statistically significant differences between groups (all adjusted *p* > 0.36).

Similarly, time to TEVAR did not differ significantly (Kruskal–Wallis H = 2.222, *p* = 0.329). Median time to TEVAR was 4.5 h (3.0–14.0) for “Neurologically Compromised”, 6.0 h (3.0–25.0) for “Shock”, and 5.5 h (3.0–16.0) for “Stable”. Dunn's *post hoc* testing again revealed no significant pairwise differences (all adjusted *p* > 0.44).

### 3.10 Explainable artificial intelligence analysis

The XGBoost surrogate classifier demonstrated high fidelity to the original K-means–derived phenotype labels. Because the supervised model was trained on the same physiologic variables used for clustering, its high classification performance was expected and is presented solely to support explainability, not predictive novelty. On the held-out test set, the model achieved an accuracy of 0.917, macro–F1 score of 0.906, Cohen's κ of 0.873, and an adjusted Rand index (ARI) of 0.788, indicating strong agreement with the unsupervised cluster structure. Five-fold cross-validation on the training set yielded similar results (mean accuracy 0.913, macro–F1 0.905, κ 0.867, ARI 0.770), supporting the generalizability of the classifier.

**Figure 7:**
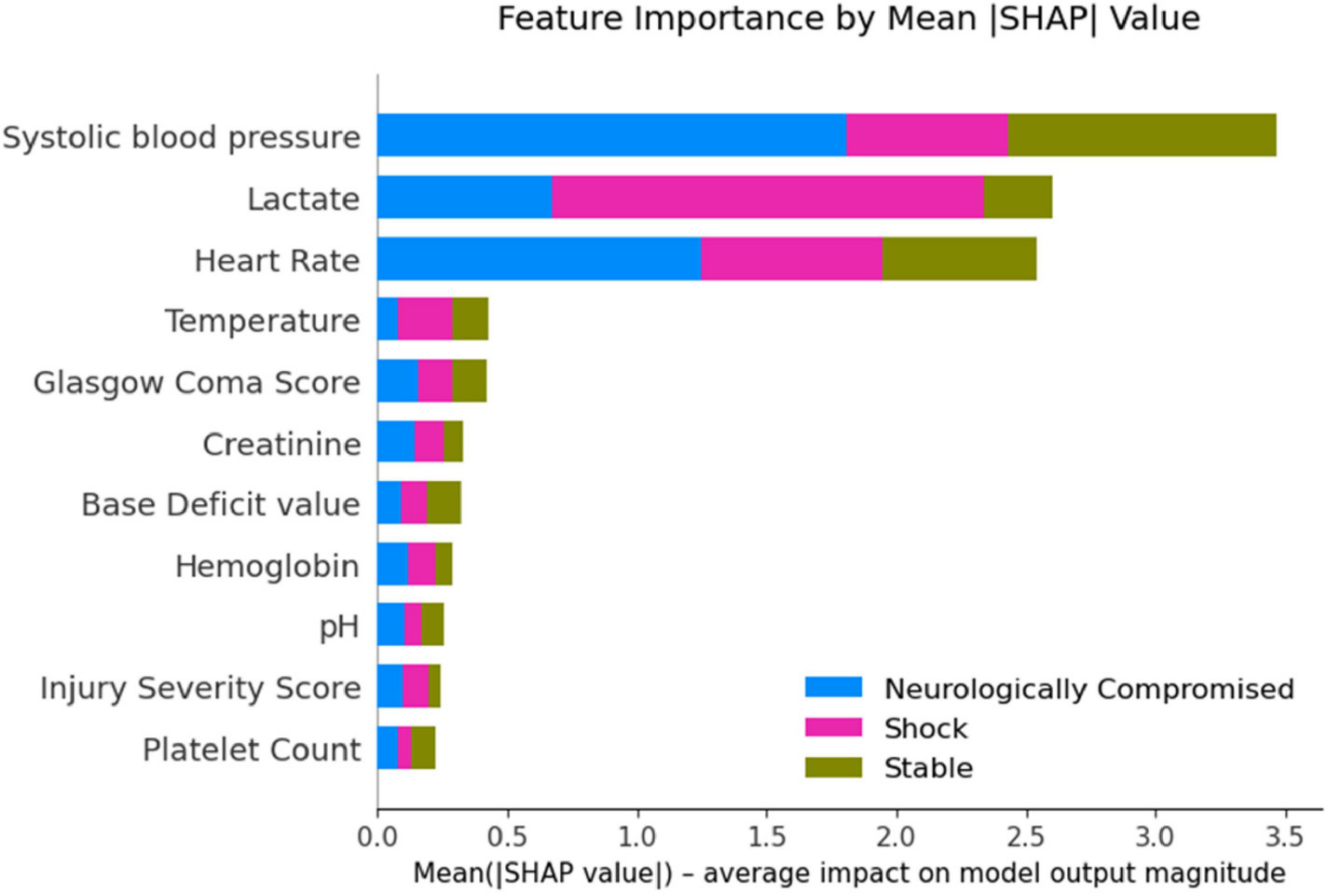
Feature importance by mean SHAP value.

Global SHAP-based feature importance (Figure 7) ranked systolic blood pressure (mean |SHAP| = 1.16), lactate (0.87), and heart rate (0.85) as the dominant physiologic drivers of phenotype assignment, followed by temperature (0.14), Glasgow Coma Score (0.14), creatinine (0.11), base deficit (0.11), hemoglobin (0.10), pH (0.08), Injury Severity Score (0.08), and platelet count (0.07). Stability analysis across 200 bootstrap refits demonstrated high reproducibility of these rankings, with a median Spearman rank correlation of 0.845 between bootstrap-derived importance orders and the original model.

**Figure 8:**
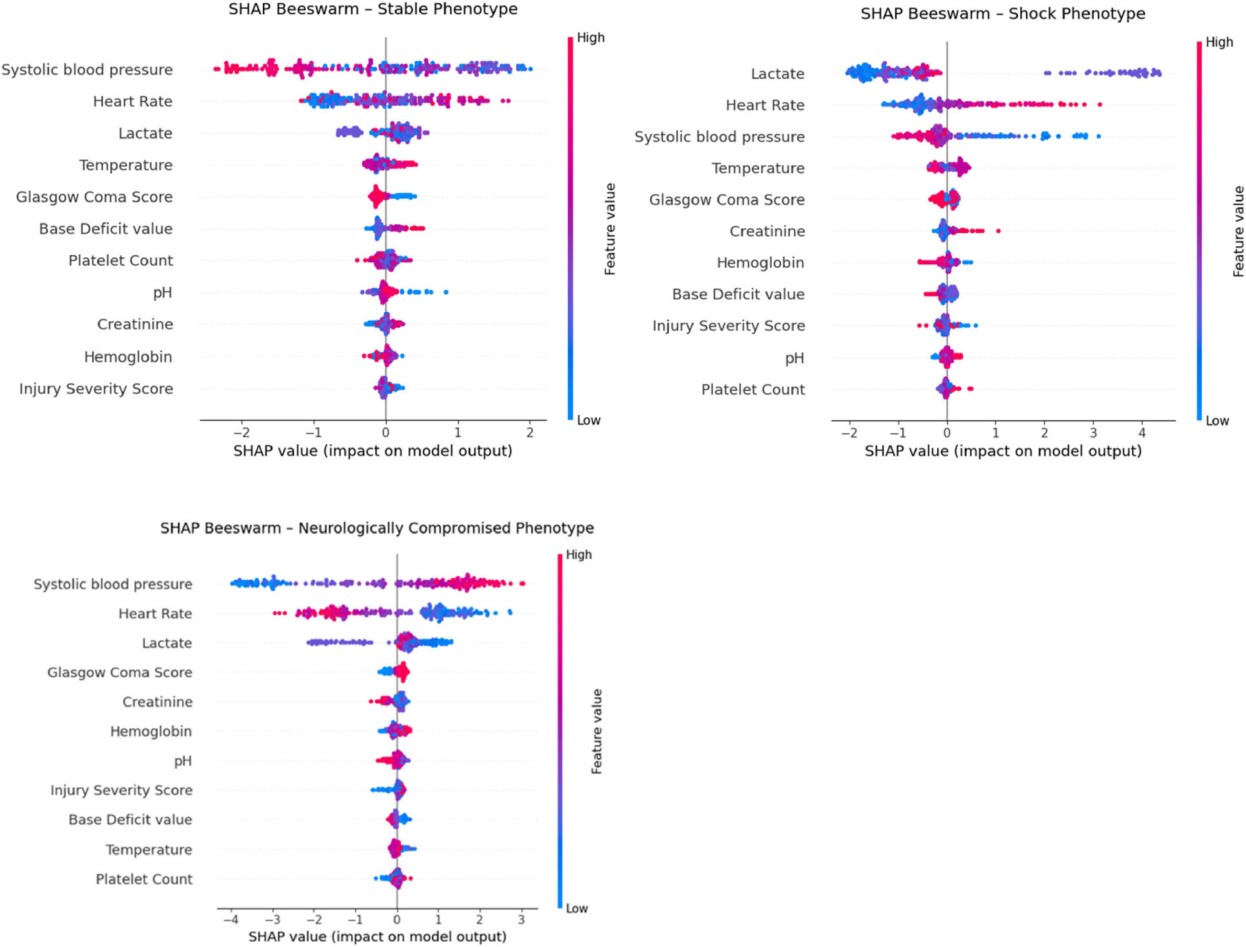
SHAP beeswarm plots demonstrating feature contributions to phenotype classification for stable (top left), shock (top right), and neurologically compromised (bottom) groups. Each point represents an individual patient, with color indicating the feature value (red = high, blue = low) and horizontal position indicating the magnitude and direction of the feature's influence on phenotype assignment.

Class-specific SHAP beeswarm plots (Figure 8) illustrated distinct physiologic signatures within each phenotype. The Shock phenotype was driven by low systolic blood pressure and high lactate and heart rate, whereas the Stable phenotype was characterized by higher blood pressure and lower lactate. The Neurologically Compromised phenotype exhibited intermediate hemodynamics with relatively higher creatinine and base deficit and modestly lower GCS, features that primarily differentiated this group from the others rather than contributing uniformly across the full cohort. Together, these XAI findings confirm that the clusters reflect physiologically coherent patterns primarily along axes of perfusion and metabolic derangement, with neurologic status providing additional within-phenotype refinement.

## 4 Discussion

In this multicenter registry study, we applied unsupervised machine learning to standardized early physiologic data and identified three reproducible admission phenotypes in patients with blunt thoracic aortic injury (BTAI): “Stable”, “Shock”, and “Neurologically Compromised”. To our knowledge, this is the first phenotyping analysis in BTAI to use purely physiologic variables rather than anatomic injury grading, and the first to employ a centroid-based approach for phenotype assignment in a refined analysis cohort. This method preserved the interpretability of the initial clustering while ensuring reproducibility under stricter data completeness requirements. These findings should be interpreted as descriptive and hypothesis-generating; this study was not designed to test causal relationships or evaluate phenotype-guided treatment strategies.

The phenotypes exhibited distinct physiologic signatures and clinically meaningful differences in mortality. The “Stable” phenotype had preserved hemodynamics, low lactate burden, and the lowest mortality, while the “Shock” phenotype reflected hypotension, tachycardia, and elevated lactate (22, 23). The “Neurologically Compromised” phenotype was defined by markedly depressed GCS with intermediate hemodynamics. Despite these different physiologic drivers of risk, mortality in both Shock and Neurologically Compromised patients was nearly threefold higher than in Stable patients. These within-phenotype mortality comparisons are descriptive and are highly confounded by clinical instability, indication bias, and early mortality, and therefore cannot be used to infer treatment effects.

Deeper statistical exploration revealed that treatment patterns, while superficially similar, differed in important ways. TEVAR was the most common modality across all phenotypes, but “Stable” patients were significantly more likely to be managed medically and less likely to have no documented intervention compared with “Shock” patients. “Shock” patients had the highest proportion of “none/unknown” interventions, which may reflect rapid deterioration or instability precluding repair. Mortality also varied markedly by modality within phenotypes. In “Shock” patients, mortality was highest for open repair and for cases without intervention, while in “Neurologically Compromised” patients, open repair mortality exceeded 75% and non-intervention mortality exceeded 50%. In contrast, “Stable” patients had low mortality across all modalities, though it was elevated in those without documented intervention. These findings suggest that physiologic phenotype interacts with treatment choice to influence outcome and that the risks and benefits of intervention may not be uniform across phenotypes.

These results generate several hypotheses. For “Shock” patients, earlier operative intervention or targeted resuscitation warrants evaluation in prospective studies, whereas in “Neurologically Compromised” patients, the high mortality with open repair should open an investigation into the need for more selective surgical decision-making, potentially delaying repair in cases of severe brain injury until prognosis is clearer. The similar mortality observed in these groups may also reflect an element of failure to rescue, underscoring the importance of timely recognition and response once physiologic deterioration occurs. Integrating phenotype-based risk stratification into existing treatment algorithms could be explored in future prospective or interventional studies, which could help refine timing, align intervention intensity with prognosis, and inform goals-of-care discussions.

Phenotyping has been successfully applied in other heterogeneous syndromes such as sepsis, ARDS, and TBI, where physiologic subgroups correlate with outcomes and, in some cases, treatment response (12–14). Applying a similar approach to BTAI is novel and may help bridge the gap between anatomical grading and physiologic complexity. The use of a data-driven clustering method with fixed centroid assignment supports reproducibility and provides a framework for prospective validation. The modest silhouette score observed in this analysis is consistent with prior physiologic phenotyping studies in heterogeneous acute care populations, where underlying biologic processes exist along continuous spectra rather than as sharply separable classes. In syndromes such as sepsis, acute respiratory distress syndrome, and traumatic brain injury, clinically meaningful phenotypes have been identified despite substantial overlap in physiologic features and modest cluster separation by unsupervised metrics (12–14, 21–23). From a methodological standpoint, silhouette values do not have absolute interpretive thresholds, and lower scores are expected in noisy, high-dimensional clinical data (18). Importantly, in this study, phenotypes demonstrated face-valid physiologic patterns, significant differences in mortality, and internally consistent feature attribution on explainable AI analysis, supporting their clinical relevance despite modest silhouette separation.

Importantly, the supervised model was used only as an interpretability scaffold and not as a validation of clinical robustness or predictive performance. The XAI analysis refined our understanding of the physiologic signatures underlying the three data-driven phenotypes and provided independent support for their validity. The XGBoost surrogate model reproduced the K-means labels with high fidelity (held-out accuracy 91.7%, macro–F1 0.906, *κ* 0.873, ARI 0.788), indicating that the phenotypes represent reproducible, learnable patterns in the physiologic data rather than artifacts of the clustering algorithm. SHAP-based feature attribution consistently highlighted systolic blood pressure, lactate, and heart rate as the most influential variables, with stable rankings across 200 bootstrap refits (median Spearman *ρ* = 0.845). Although the Neurologically Compromised group was initially labeled based on depressed GCS, the explainability analysis showed that neurologic status contributed less to global cluster separation than perfusion-related variables, suggesting that this phenotype reflects a mixed picture of impaired consciousness in the setting of moderate hypoperfusion and metabolic stress. Rather than undermining the construct, this pattern emphasizes that the phenotypes capture integrated cardiocirculatory and neurologic dysfunction, aligning with recognizable clinical constructs and strengthening the biologic plausibility of the unsupervised clusters.

Importantly, this does not negate the validity of the phenotype; instead, it highlights that its pathophysiology is integrative, combining both central and systemic dysfunction. The lower global SHAP weight for GCS reflects that it distinguishes one cluster from the others, but is not uniformly informative across all groups, consistent with the clinical heterogeneity of traumatic aortic injury patients.

These explainability findings strengthen the biologic plausibility of the unsupervised phenotypes by demonstrating that their defining axes correspond to recognizable physiologic constructs: circulatory stability (Stable), systemic hypoperfusion (Shock), and combined neurologic–metabolic compromise (Neurologically Compromised).

Limitations include the retrospective design, single-time-point phenotyping, and the possibility that physiologic patterns reflect associated injuries rather than the aortic injury itself. The study cohort demonstrated a marked male predominance, consistent with prior epidemiologic studies of blunt thoracic aortic injury, which disproportionately affects young and middle-aged men involved in high-energy mechanisms such as motor vehicle collisions (24). While this imbalance reflects real-world injury patterns rather than sampling bias, it may limit the generalizability of the identified phenotypes to female patients. Nonetheless, the observed interaction between phenotype, treatment modality, and mortality underscores the potential value of physiology-informed decision-making in BTAI. Future studies should validate these phenotypes prospectively, explore their temporal stability, and test whether phenotype-guided treatment strategies can improve outcomes.

## 5 Conclusion

In this multicenter registry analysis, unsupervised machine learning identified three reproducible physiologic phenotypes among patients with blunt thoracic aortic injury: Stable, Shock, and Neurologically Compromised. These phenotypes demonstrated distinct admission physiologic profiles and significantly different in-hospital mortality, despite broadly similar treatment patterns. *post hoc* explainable AI analysis showed that perfusion- and metabolism-related variables, particularly systolic blood pressure, lactate, and heart rate, were the dominant drivers of phenotype assignment, with neurologic status contributing primarily within a single subgroup.

Together, these findings highlight the heterogeneity of early physiologic presentation in BTAI and suggest that anatomy alone may be insufficient for risk stratification. While this study is descriptive and hypothesis-generating, it provides a foundation for future prospective work to evaluate whether physiologic phenotyping can inform prognostication, triage, and timing of intervention in this high-risk population.

## Data Availability

The data analyzed in this study is subject to the following licenses/restrictions: Permission was obtained by the Aortic Trauma Foundation for the use of this dataset. Requests to access these datasets should be directed to https://aortictrauma.org.

## Aortic trauma foundation investigators

Naveed Saqib, Anthony Estrera, Gustavo Oderich, Rana Afifi, Hong Quang Le, Michael Lee, Michelle McNutt, Dave Meyer, Binod Shrestha, Edmundo Dipasupil, Elina Quiroga, Nicolas Stafforini, Charles Fox, Rishi Kundi, Rami Gilani, Jorge Miranda, David Turay, Xian Luo-Owen, Muhammad Aftab, Mohammed Al-Musawi, T. Brett Reece, Donald Jacobs, Rafael D. Malgor, Jeniann Yi, Erica Leith Mitchell, Martin Croce, Suzanne Moyer, Lalithapriya (Priya) Jayakumar, Matthew J Sideman, Christopher Mitromaras, Dimitrios Miserlis, Reshma Brahmbhatt, Lisa Bennett, Ernest Moore, Alexis Cralley, William Shutze, William Dockery, Laura Petrey, Timothy N Phelps, Nicole Fox, Lisa Shea, John Berne, Ivan Puente, Mario F Gomez, Dalier R Mederos, Pedro Teixeira, Emily Leede, Frank Buchanan, Emilio Ramos, Marielle Ngoue, Sadia Ali, Davide Pacini, Luca Botta, Ciro Amodio, Tal Horer, David McGreevy, Ravi Rajani, Jaime Benarroch-Gampel, Christopher R. Ramos, Manuel Garcia-Toca, Kenji Inaba, Matt Smeds, Emad Zakhary, Michael Williams, Catherine Wittgen, Pierantonio Rimoldi, Ilenia D'Alessio, Nicola Monzio Compagnoni, Valerio Tolva, Neil Parry, Luc Dubois, John Bini, Karen Herzing, Marc Passman, Adam W Beck, Mark Patterson, Ben Pearce, Emily Spangler, Jarrad Rowse, Danielle Sutzko, Andres Schanzer, Francesco Aiello, Edward Arous, Elias Arous, Douglas Jones, Dejah Judelson, Louis Messina, Tammy Nguyen, Jessica Simons, Robert Steppacher, James Haan, Kelly Lightwine, Vincent Riambau, Gaspar Mestres, Xavier Yugueros, Daniel Gil, Eduard Casajuana, Malachi Sheahan, Marie Unruh, Claudie Sheahan, Tapash Palit, Amit Chawla, Amadis Brooke, Melissa Donovan, Joe Giaimo, Bruce Torrance, Joao Rezende-Neto, Mario D'Oria, Sandro Lepidi, Peter Rossi, Viktor Reva, Trissa Babrowski, Ross Milner, Luka Pocivavsek, Christopher Skelly, Julie Dunn, Brittany Smoot, Sam Godin, Kevin Martin, Todd Vogel, Santi Trimarchi, Maurizio Domanin, Viviana Grassi, Matt Eagleton, Ali Azizzadeh, Bruce Gewertz, Galinos Barmparas, Donald Baril, Elizabeth Chou, Cassra Arbabi, NavYash Gupta, Sally Schonefeld, Theodore Teruya, Marc Schermerhorn, Mark Wyers, Allen Hamdan, Lars Stangenberg, Andy Lee, Patric Liang, Christina Marcaccio, Junaid Malek, Giovanni Ferrante, Emmanuel Nwachuku, Ralph Darling, Xzabia Caliste, Benjamin B. Chang, Jeffrey C. Hnath, Paul B. Kreienberg, Alexander Kryszuk, Adriana Laser, Sean P. Roddy, Stephanie Saltzberg, Melissa Shah, Courtney Warner, Chin-Chin Yeh, Allison Berndtson, Asad Choudhry, Joseph Galante, Lee Seong, Seong K. Lee, Andrew Rosenthal, Rachele Solomon, Sergi Bellmunt, Robert Rhee, Susan Beale, Lewis E. Jacobson, Jamie Williams, Christopher Firek, Xiaofei Zhang, Alex Coronel, Megan Brenner, Zachary Wanken, Jahanzeb Kaikaus, Daniel Kindell, Richie Li, Erin McIntosh, Fatima Mustansir, Julia Suggs, Shirli Tay, Varun Dalmia, Ryan Wahidi, Muhammad Zeeshan, Henry Jefferson, Heather Grossman Verner, Cynthia Villalta, Rene Chidozie, R Pulli, “Rossella Di Domenico, Sara Speziali”, Giorgio Turicchia, Mara Fanelli, Manar Khasram.
